# Physicochemical Properties of Various 2-Hydroxyethylammonium Sulfonate -Based Protic Ionic Liquids and Their Potential Application in Hydrodeoxygenation

**DOI:** 10.3389/fchem.2019.00196

**Published:** 2019-04-05

**Authors:** Guangming Cai, Shaoqi Yang, Qing Zhou, Lifei Liu, Xingmei Lu, Junli Xu, Suojiang Zhang

**Affiliations:** ^1^Beijing Key Laboratory of Ionic Liquids Clean Process, CAS Key Laboratory of Green Process and Engineering, State Key Laboratory of Multiphase Complex Systems, Institute of Process Engineering, Chinese Academy of Sciences, Beijing, China; ^2^School of Chemical Engineering, University of Chinese Academy of Sciences, Beijing, China

**Keywords:** protic ionic liquids, density, viscosity, electrical conductivity, dissociation constants, deoxidation reaction

## Abstract

In order to obtain the regularities of physicochemical properties of hydroxy protic ionic liquids (PILs) and broaden their potential application, a series of 2-hydroxyethylammonium sulfonate-based PILs were synthesized through proton transfer reaction and characterized by NMR and FT-IR and elemental analysis. Their phase transfer behavior (*T*_m_) and initial decomposition point (*T*_d_) were characterized by differential scanning calorimetry (DSC) and thermogravimetric analysis (TGA), respectively. Meanwhile, the regularities of density (ρ), viscosity (η) and electrical conductivity (σ) of synthesized PILs at different temperatures were measured. The results indicated that their physicochemical properties were tightly related with their structures and the interactions between cations and anions. In addition, the dissociation constants (p*K*a) of synthesized PILs were obtained by acid-base titration, which revealed that all synthesized PILs had p*K*a exceeding 7 and their cations were the crux of determining the p*K*a value. Moreover, several synthesized PILs with a low melting temperature also showed potential application in the deoxidation reaction of cyclohexanol, as they had conversion rates approximating 100% and the selectivity of cyclohexane or cyclohexene was about 80%.

## Introduction

Due to their unique and tunable characteristics, like low vapor pressure, high thermal stability, high conductivity and low flammability, ionic liquids (ILs), as a kind of functionalized solvents, have gained a lot of attention around the world (Wang et al., 2016; Miran et al., 2018; Xia et al., 2018; Yuan et al., 2018). In general, ILs can be classified in two categories, aprotic and protic ionic liquids (PILs), according to their structure characteristics (Greaves and Drummond, 2015; Shang et al., 2017). Among them, PILs are prepared by the neutralization reaction of certain Brønsted acids and Brønsted bases (generally from primary, secondary or tertiary amines, which are alkaline) and the fundamental feature of these kinds of ILs is that their cations have at least one available proton to form hydrogen bond with anions (Vijayraghavan et al., 2013; Greaves and Drummond, 2015). In addition to the above aspects, the biggest advantage of PILs is the low-cost and simple synthetic procedure, which means that there is a great potential for large-scale applications (Brandt-Talbot et al., 2017; Sun et al., 2017). In recent years, PILs have been widely applied in electrochemistry (Armand et al., 2009; Vogl et al., 2014), natural products extraction (Tang et al., 2012), liquid-liquid extractions and separation (Elshwishin et al., 2014), gas capture (Shang et al., 2017), biomass processing (Elgharbawy et al., 2016; Brandt-Talbot et al., 2017; Sun et al., 2017; Yang et al., 2018), as well as catalytic processes (Vancov et al., 2012; Vekariya, 2017).

In order to meet the experimental or practical requirements, many attempts have been made to synthesize PILs with different anions and cations according to the desired properties (Brandt-Talbot et al., 2017; Shang et al., 2017; Sun et al., 2017; Qu et al., 2018; Yang et al., 2018). In this process, the type of PILs with hydroxy cation increasingly draws attention from academic and industrial application fields, because the hydroxy group in ILs facilitates the formation of strong hydrogen bonds between PILs and various substrates (Greaves and Drummond, 2015; Yang et al., 2018). This feature promotes hydroxy PILs to be applied in many fields. For example, S. Yang et al. used the hydroxy PILs to pretreat lignocelluloses and the results showed that PILs displayed good efficiency for extracting cellulose from corn straw (Yang et al., 2018). J. Sun et al. demonstrated that ethanolamine acetate was effective to produce cellulosic ethanol from switch grass in one-pot process (Sun et al., 2017). In addition to the consideration of cationic species, anions are also significant in the process of designing PILs in that the anions of PILs, as a kind of deprotonated acids, play crucial roles in many fields, like catalysis, gas absorption as well as biomass pretreatment and so on (Latos et al., 2018; Ren et al., 2018). Therefore, the combination of acidic anions and basic cations with various functional groups of PILs foreshadows a lot of meaningful and useful properties, which can widen potential applications of this type of PILs.

In recent years, a large number of hydroxy PILs have been reported in the literature along with their potential application (Sun et al., 2017; Ren et al., 2018; Yang et al., 2018). However, there is little exploration between molecular structures and physicochemical properties about hydroxy PILs, which has limited the further predication of physicochemical properties for this type of PILs (Xuedan Song et al., 2012). In this work, a series of 2-hydroxyethylammonium sulfonate-based PILs are synthesized and characterized by NMR, FT-IR, TGA, and DSC systematically. Also, their physicochemical properties, such as density (ρ), viscosity (η), electrical conductivity (σ) and acid-base property (p*K*a) are measured by density meter, viscometer, conductivity meter and acid-base titration, respectively. Moreover, the regularities of physicochemical properties about the PILs are explained through molecular structures and the interactions between cations and anions, which is helpful to predict the change law of similar PILs in the future. Furthermore, the deoxygenation is a very important reaction during lignin hydrodeoxygenation (HDO) processes (Yan et al., 2010; Chen et al., 2016, 2017). These reactions are mostly catalyzed by using noble metal catalysts or protic acids according to the reported methods (Güvenatam et al., 2014). Herein, in order to explore the applicability of the synthesized ILs, several PILs with a low melting temperature are used as the potential catalysts for the deoxygenation reaction with cyclohexanol as the substrate.

## Experimental Section

### Materials

The PILs presented in this manuscript were synthesized following the procedures described in the next section. N-methyldiethanolamine (>99%), N, N-dimethylethanolamine (>99%), triethanolamine (>99%), methanesulfonic acid (>99%), trifluoromethanesulfonic acid (>98%), benzenesulfonic acid (>98%), dodecane (GC, ≥99.0%), cyclohexanol (GC, >99.5%), cyclohexene (GC, >99.5%), cyclohexane (GC, >99.5%), and methyl tert-butyl ether (>99.5%) were from Aladdin Chemistry Co., Ltd. and were used as received. Ethanolamine (>99%) and diethanolamine (>99%) were from Xilong Chemical Co., Ltd. Ethyl acetate (>99.5%), ether (>99.5%) and methanol (>99.5%) were purchased from Sinopharm Chemical Reagent Co., Ltd. H_2_ was provided by the Beijing Beiwen Gas Factory, and the purity was 99.999%.

### Synthesis Procedures

**Figure 1:**
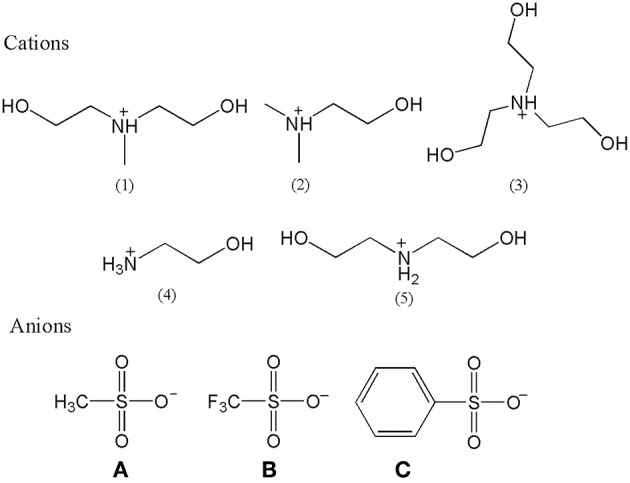
The cations and anions structures of synthetized PILs.

All PILs were synthesized according to the same reaction mechanism, which was the neutralization reaction of Brønsted acids and Brønsted bases. Other references had reported the relevant synthesis procedures with similar structures (Xuedan Song et al., 2012; Cao et al., 2015; Hosseini et al., 2018), but the operation procedures were slightly different. The relevant structure of cations and anions of synthesized PILs are shown in Figure 1 and their description and abbreviation are presented in Table 1. Among them, [MDEA][mesy], [DMEA][mesy], [TEA][mesy], [TEA][OTf], [TEA][Bsa], [ETA][OTf], and [DEA][OTf] have been reported in other studies (Xuedan Song et al., 2012; Gruzdev et al., 2017; Yang et al., 2018). The remaining PILs were reported for the first time in this study.

**Table 1:** The description and abbreviation of synthesized PILs.

**No**.	**Description**	**Abbreviation**
1a	N-methyl- Bis(2-hydroxyethyl)ammonium methanesulfonate	[MDEA][mesy]
2a	N,N-dimethyl-2-hydroxyethylammonium methanesulfonate	[DMEA][mesy]
3a	Tris(2-hydroxyethyl)ammonium methanesulfonate	[TEA][mesy]
4a	2-hydroxyethylammonium methanesulfonate	[ETA][mesy]
5a	Bis(2-hydroxyethyl)ammonium methanesulfonate	[DEA][mesy]
1b	N-methyl- Bis(2-hydroxyethyl)ammonium trifluoromethanesulfonate	[MDEA][OTf]
2b	N,N-dimethyl-2-hydroxyethylammonium trifluoromethanesulfonate	[DMEA][OTf]
3b	Tris(2-hydroxyethyl)ammonium trifluoromethanesulfonate	[TEA][OTf]
4b	2-hydroxyethylammonium trifluoromethanesulfonate	[ETA][OTf]
5b	Bis(2-hydroxyethyl)ammonium trifluoromethanesulfonate	[DEA][OTf]
1c	N-methyl- Bis(2-hydroxyethyl)ammonium benzenesulfonate	[MDEA][Bsa]
2c	N,N-dimethyl-2-hydroxyethylammonium benzenesulfonate	[DMEA][Bsa]
3c	Tris(2-hydroxyethyl)ammonium benzenesulfonate	[TEA][Bsa]
4c	2-hydroxyethylammonium benzenesulfonate	[ETA][Bsa]
5c	Bis(2-hydroxyethyl)ammonium benzenesulfonate	[DEA][Bsa]

### Synthesis of PILs Containing Methanesulfonate

A 500 mL one-neck flask and a 250 mL constant pressure dropping funnel were used to assemble the reaction apparatus. Using 200 mL methanol as the solvent, 0.1 mol of methanesulfonic acid was added in the equimolar alkanolamine solution (N-methyldiethanolamine, N, N-dimethylethanolamine, triethanolamine, ethanolamine, and diethanolamine) drop by drop in the condition of ice bath stirring (at 600 rpm) with a magnetic stirrer (**IKA**® **C-MAG** HS 7). The reaction was acted at room temperature for 48 h. Then, the methanol was removed by rotary evaporation (RE-2000E, Beijing Xingde Instrument Equipment Co., Ltd.) at 40°C and vacuum degree of 0.1 MPa for 2 h, thus the corresponding PILs were obtained. 200 mL Ethyl acetate and ether were used to wash the PILs three times, respectively. Then all PILs were dried out in a vacuum drying oven (DZG-6021, Shanghai Sumsung Laboratory Instrument Co., Ltd.) in the condition of water absorbent P_2_O_5_ at 60°C and vacuum degree of 0.1 MPa for 120 h. To maintain high water absorption, P_2_O_5_ was replaced every 10 h.

### Synthesis of PILs Containing Trifluoromethanesulphonate and Benzenesulfonate

As trifluoromethanesulfonic acid could easily absorb water in the air, the agent was quickly transferred and reacted in the glove box. Also, before benzenesulfonic acid was used, 0.1 mol benzenesulfonic acid was dissolved in 100 mL of methanol to make the solution, because the acid was in a solid state at room temperature. The remaining procedures were similar to the procedures of the synthesis of methanesulfonate PILs.

### Instrumental and Operation Procedures

#### NMR and FT-IR Spectroscopy as Well as CHNS Analysis

The synthesized PILs were characterized by ^1^H and ^13^C NMR (AVANCE III HD-600 MHz, Bruker, Switzerland) in deuterated dimethyl sulfoxide (DMSO-*d*_6_) with reference of TMS. FT-IR (10,000–370 cm^−1^, Thermo Nicolet 380 spectrometer, America) was used to verify the chemical bond vibration peaks of synthesized PILs. The range of scanning wavelength was 4,000–400 cm^−1^ at a repeat of 32 times with a KBr tablet. An elementar vario EL cube was used for the elemental analysis. Before the measurement, each PIL (2–3 mg) was capsuled in tin capsules (volume 0.05 mL) with three parallel samples. The final results were obtained by calculating the arithmetic mean values of the three samples.

#### Water Content

The water content of synthesized PILs were determined by Karl Fischer titrator (METTLER TOLEDO, C20 Coulometric KF Titrator). Before water measurement, the titrator was turned on and ran the baseline to make the water content balance at about 10 ppm. Approximately 3 mg PILs sample was then pipetted into the instrument using a plastic dropper to begin moisture measurements. The water was measured immediately after PILs were dried out in vacuum drying oven for 120 h.

#### Thermogravimetric Analysis (TGA)

TG/DTA6300 (Hitachi, JP) was used to get the TGA curves. About 15 mg PILs samples were heated in the TGA crucible from room temperature to 100°C at a rate of 10°C/min at flow of 50 mL/min N_2_ carrier first. It was set to remove any possible volatile impurities (such as water, washing solvents) in the PILs. When the crucible cooled to room temperatures again, the sample was heated to 800°C under the same conditions. The instrument recorded the weight residue percentage automatically.

#### Differential Scanning Calorimetry (DSC)

Differential scanning calorimetry (DSC) was performed using METTLER TOLEDO DSC 1 S**TA**R^e^ System in a sealed aluminum pan under nitrogen atmosphere (50 mL/min). Ultramicro electronic balance (METTLER TOLEDO, XS105 DualRange) was used to weigh about 5 mg PILs. Temperature programming was set from 25 to 100°C at a heating rate of 10°C min^−1^, then keeping 10 min to evaporate the remaining water or any volatile impurities in the sample. Finally, the temperature was decreased to −50°C and from there, the temperature programming started to increase from −50 to 350°C at the same heating rate.

### Density (ρ) and Viscosity (η) Measurement

The density and viscosity of synthesized PILs were measured by an all-in-one machine comprised of Anton Paar DMA™ 5000 M density meter and Anton Paar micro viscometer Lovis 2000 ME at temperature (293.15–353.15) K. The density meter's accuracy is ± 0.000007 g/cm^3^ and the temperature in density chamber can accurate to 0.01 K. The micro viscometer's accuracy can up to ± 0.5% in the experiment and the temperature of glass capillaries can precise to ± 0.02 K. There are three kinds of glass capillaries (1.59, 1.8, 2.5) mm with viscosity range of (0.3–15,10–100 and 100–10,000) mPa·s, respectively. The capillaries were calibrated by Anton Paar company before using. All samples were degassed in the condition of 320 K before measuring. Every point was repeated at least three times and the calculated average was used as the final data.

### Electrical Conductivity (σ)

The conductivity of all PILs samples was measured by conductivity meter (METTLER TOLEDO FiveEasy Plus) at the temperature range from (303.15 to 343.15) K. The maximum operating temperature of the conductivity cell was 353.15 K due to restrictions imposed by its plastic framework. Before measuring, the electrode of conductivity meter was calibrated using the standard solutions.

### Thermodynamic Dissociation Constants (p*K*_a_) Determination

The standard method of determining the p*K*a of substance was described in the literature (Chen et al., 2014). The p*K*a values of various PILs were obtained by acid–base titration. The specific operation steps were listed in the Supplementary Material. The dissociation constant was expressed as follows:

(1) $$K_{a}=\frac{\left[{\text{Na}}^{\text{+}}\right]\text{+}\left[{\text{SH}}_{\text{2}}^{\text{+}}\right]}{[{\text{MSO}}_{\text{3}}^{-}]-\left[{\text{Na}}^{\text{+}}\right]-\left[{\text{SH}}_{\text{2}}^{\text{+}}\right]}*\left[{\text{SH}}_{\text{2}}^{\text{+}}\right]$$

where [X] stands for the concentration of each ion at any moment of titration and the$\left[{\text{MSO}}_{3}^{-}\right]$ stands for the concentration of anion of each PIL in the experiments. The Equation (1) is applicable when the initial pH of solution is <7 and the only unknown value is [${\text{SH}}_{2}^{+}$]. In order to obtain the value of the unknown [${\text{SH}}_{2}^{+}$], the following equation was used to confirm its value:

(2) $$[{\text{SH}}_{\text{2}}^{\text{+}}]=\frac{a\left[{\text{SH}}_{\text{2}}^{\text{+}}\right]}{f\left[{\text{SH}}_{\text{2}}^{\text{+}}\right]}$$

where $f\left[{\text{SH}}_{2}^{+}\right]$is the activity coefficient and $a\left[{\text{SH}}_{2}^{+}\right]$ is the activity of $\left[{\text{SH}}_{2}^{+}\right]$. By using a series of volume and concentration data of titrant and titrand, the value of p*K*a could be calculated. A more detailed derivation process can be seen in the Supplementary Material.

### Catalytic Reaction for the Deoxidation of Cyclohexanol

The chemicals of cyclohexanol (1 mmol), ionic liquid (2 g) and n-dodecane (1 mmol) were added into an autoclave with a Teflon® cell. The reaction system was sealed and purged with high-purity hydrogen for at least three times in order to get the air out of the cell. Then the autoclave was pressurized with 4 MPa H_2_ at room temperature. The autoclave was heated from room temperature to 120°C. When the temperature rose to the set point, the agitator was opened about 600 rpm and timing started for 6 h. After the reaction finished, the autoclave was cooled in ice bath for about 2 h. The final products were extracted by 8 ml methyl tert-butyl ether (MBTE) and analyzed through gas chromatography mass spectrometry (GC-MS, Shimadzu GCMS-QP2020), and quantified with gas chromatography equipment (GC-2014, Shimadzu). The GC system was equipped with a capillary column from Agilent (RTX-5®, 30 m × 0.25 mm × 0.25 μm) connecting flame ionization detector (FID) for quantification. N-dodecane was used as the internal standard in the experiments.

## Results and Discussion

### Characterization of Synthesized PILs

The ^1^H and ^13^C NMR spectra are presented in Figures S1–S15. From the spectra, all H and C atoms shifts and peaks corresponded to the structures of PILs with reference of TMS. Meanwhile, the areas of peak were proportionable with the number of H atoms in the PILs. CHNS analysis indicated that the proportion of each element in synthesized PILs was consistent with the calculated values. The specific found values of CHNS analysis are listed in the Supplementary Material. In addition, all infrared spectra of explored PILs are presented in Figures S16–S30 in the Supplementary Material and the infrared absorption peaks corresponded to the corresponding groups. What's more, The Karl Fischer titration results indicated that water was about (1,000–3,000) ppm for most part of samples. The specific water content of all samples are presented in Table S1 in the Supplementary Material. Combining the analysis of ^1^H NMR, ^13^C NMR, CHNS analysis and FT-IR spectra as well as Karl Fischer titration and elemental analysis, the purity of PILs containing methanesulfonate and trifluoromethanesulphonate was more than 99% and those containing benzenesulfonate exceeded 98%.

### TGA and DSC Analysis

Thermogravimetric analysis (TGA) is an available way to characterize the thermostability of synthesized PILs and get their initial decomposition temperature (*T*_d_) as well as to ascertain their feasible working temperature range. The regularities of weight loss of various PILs with the increase of temperature are shown in Figure S31 and the temperature at 5 % weight loss of sample was identified as *T*_d_ (Castro et al., 2016; Huang et al., 2018). The *T*_d_ of all synthesized PILs are presented in Table 2. Among them, [ETA][OTf] had the highest *T*_d_ (314°C) and [DMEA][mesy] had the lowest one (156°C). In addition, it was noteworthy that the *T*_d_ was closely related to cations. In other words, PILs with the same cation have similar *T*_d_. For example, the *T*_d_ of [TEA][mesy], [TEA][OTf] and [TEA][Bsa] were 266, 278, and 260°C, respectively. However, the *T*_d_ of [MDEA][mesy], [DMEA][mesy], [TEA][mesy], [ETA][mesy], and [DEA][mesy] were 205, 156, 266, 286, and 289°C, that varied greatly. Also, for the majority of synthesized PILs, they were degraded step by step as shown in Figure S31. The most probable reason for this phenomenon was that the thermostability of anions and cations were different because they consisted of different groups. For example, for the cation containing hydroxyl, it was very easy to dehydrate at high temperature. Therefore, with the increase of temperature, the components that were thermal unstable degraded first and the components that were thermal stable decomposed later (Maton et al., 2013, Venkatraman and Alsberg, 2016).

**Table 2:** The melting point (*T*_m_) and initial decomposition temperature (*T*_d_) of synthesized PILs.

**PILs**	***T*_m_ (°C)^a^**	***T*_d_ (°C)^b^**
[MDEA][mesy]	< −50	205
[DMEA][mesy]	112	156
[TEA][mesy]	85	266
[ETA][mesy]	102	286
[DEA][mesy]	< − 50	289
[MDEA][OTf]	< −50	225
[DMEA][OTf]	37	167
[TEA][OTf]	< −50	278
[ETA][OTf]	85	314
[DEA][OTf]	26	305
[MDEA][Bsa]	< − 50	238
[DMEA][Bsa]	< −50	284
[TEA][Bsa]	−32	260
[ETA][Bsa]	96	310
[DEA][Bsa]	−39	294

a *The melting point (T_m_) of PILs was determined by the maximum endothermic peak of the DSC curve before the decomposition peak*.

b *The initial decomposition temperature of PILs was confirmed by the decomposition temperature at 5% loss of weight of samples*.

Except for that, the melting points (*T*_m_) of various PILs were explored making use of the DSC curve. The curves are shown in Figure S32 in the Supplementary Material and the *T*_m_ are presented in Table 2. Table 2 clearly shows that the *T*_m_ of most of synthesized PILs were below 0°C and only [DMEA][mesy] and [ETA][mesy] had *T*_m_ of more than 100°C. In addition, it was not hard to find that PILs containing ethanolamine cation had high *T*_m_ because Table 2 shows that the *T*_m_ of [ETA][mesy], [ETA][OTf], and [ETA][Bsa] was 102, 85, and 96°C, respectively. The possible reason for this phenomenon might be that ethanolamine was a small cation compared to others and that lead to strong interactions between cations and anions, which were crucial in the process of determining *T*_m_ (Alan et al., 2001; Kireeva et al., 2012). Based on the discussion of *T*_d_ and *T*_m_, it was found that the working temperature range, namely the range of being liquid and stable state, were wide for most of synthesized PILs. This property was helpful to the potential application in the field requiring high temperature.

### Density (ρ)

As several PILs were in a solid state at room temperature, Table S2 only summarizes the density data of 10 PILs which were in a liquid state at room temperature. In order to evaluate the influence of temperature to density of synthesized PILs, the following equation was used to characterize the change law of density with the increase of temperature (Khan et al., 2017; Chen et al., 2018; Prasad et al., 2018b):

(3) $$\begin{array}{l} \rho =A^{*}T+B \end{array}$$

where ρ stands for the density of PILs at specific temperature, *T* represents the Kelvin temperature, parameter *A* is the coefficient of density as a function of temperature and *B* is a constant.

**Figure 2:**
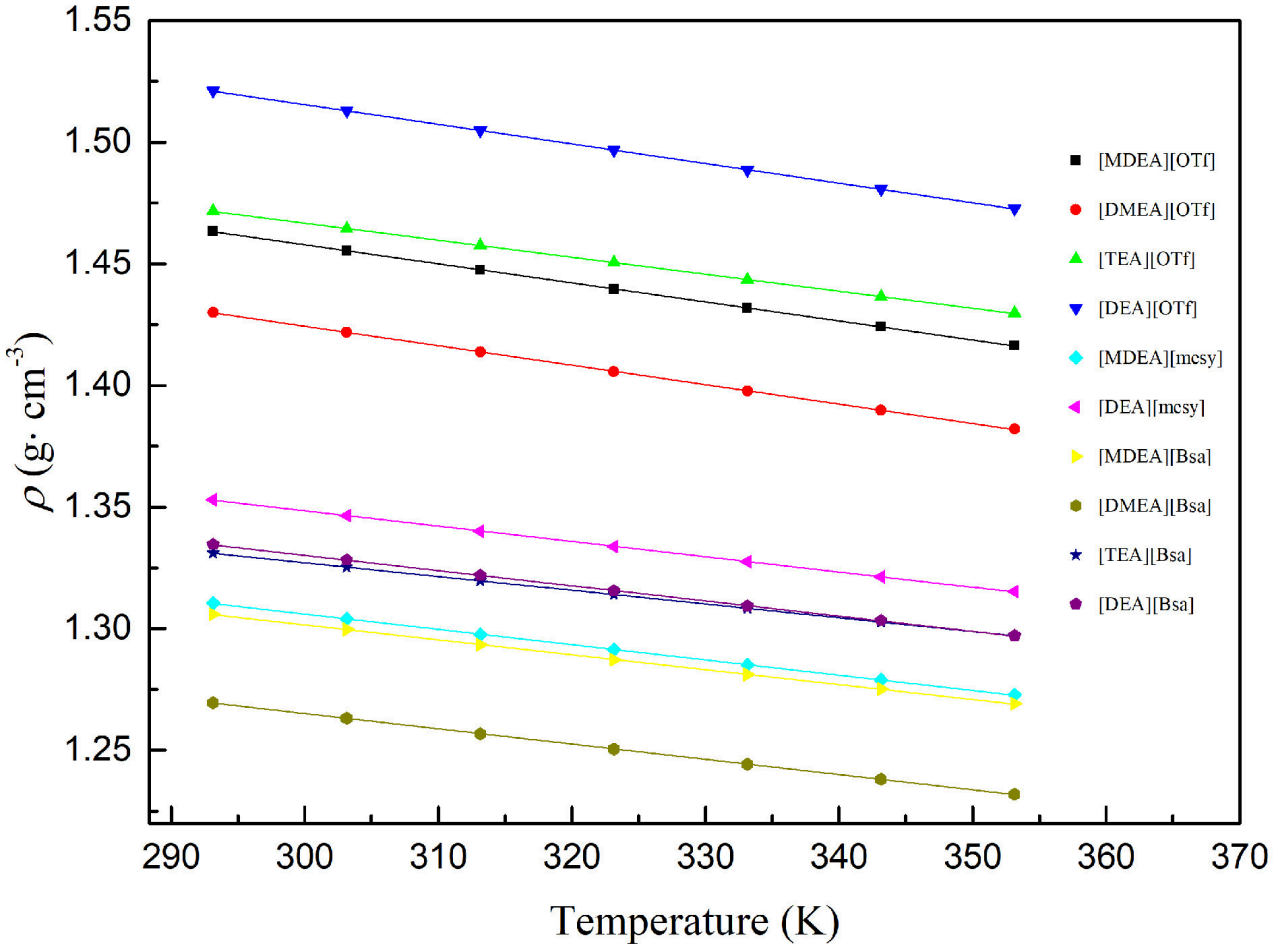
Density of various PILs at temperature (293.15 to 353.15) K. the dots are experimental density data and colored lines are fitting curves according to formula (3).

Table 3 summarizes the coefficient *A* and constant *B* as well as the correlation coefficient R^2^, which clearly indicated that the linearity of the curves was good, R^2^ for all samples were approaching 1. Also, the fitting curves are shown in Figure 2 and the change law of density as well as their distribution can be observed. Specifically, PILs with the anion of trifluoromethane sulfonate had a higher density. However, the density of PILs containing anions of benzene ring and methane sulfonate had overlaps to some extent. In addition, when synthesized PILs had the same anion, the PILs containing the cation of diethanolamine had the highest density, but those containing the cation of dimethylethanolamine had the lowest density. As the density of compound was codetermined by the molar mass as well as the molar volume (closely related with the structures of ions), PILs with the same anion and different cations would have different densities due to the different structures of cations.

**Table 3:** Coefficient *A* and constant *B* as well as correlation coefficient R^2^ of formula (3).

**PILs**	***A* (g·cm^−3^·K^−1^)**	***B* (g·cm^**−3**^)**	***R^**2**^***
[MDEA][OTf]	−7.83E-04	1.6927	0.9999
[DMEA][OTf]	−8.00E-04	1.6643	0.9999
[TEA][OTf]	−6.99E-04	1.6765	1.0000
[DEA][OTf]	−8.07E-04	1.7575	0.9999
[MDEA][mesy]	−6.28E-04	1.4943	0.9999
[DEA][mesy])	−6.30E-04	1.5374	0.9999
[MDEA][Bsa])	−6.13E-04	1.4855	0.9999
[DMEA][Bsa]	−6.27E-04	1.4532	0.9999
[TEA][Bsa]	−5.67E-04	1.4973	1.0000
[DEA][Bsa]	−6.27E-04	1.5182	0.9999

The thermal expansion coefficient (α_*P*_) was calculated using the density data through the following equation: (Khan et al., 2017; Prasad et al., 2018a; Sayah et al., 2018)

(4) $$\begin{array}{l} \alpha_{P}=-\frac{1}{\rho }\;{\left(\frac{\partial \rho }{\partial \text{T}}\right)}_{\text{P}}=-\frac{A}{AT+B} \end{array}$$

where ρ represents the density of PILs at specific temperature and pressure, *T* is the Kelvin temperature, *A* and *B* are parameters calculated by the above function (3). The calculated α_*P*_ of different PILs at investigated temperatures from (293.15 to 353.15) K are summarized in Table 4. The increasing trend of α_*P*_ of PILs with the increase of temperature could be observed, which indicated that density of PILs changed more dramatically at a higher temperature. Among those PILs, [DMEA][OTf] had the highest thermal expansion coefficient from 5.59 ^*^10^−4^ K^−1^ at 293.15 K to 5.79 ^*^10^−4^ K^−1^ at 353.15 K and [TEA][Bsa] had the lowest ones from 4.26 ^*^10^−4^ K^−1^ to 4.37 ^*^10^−4^ K^−1^ at the same temperature range. In addition, when PILs had the same anion, they had the lowest α_*P*_ in the case of containing triethanolamine and the highest α_*P*_ in the case of containing dimethylethanolamine, such as α_*P*_:[*DMEA*][*OTf*] > [TEA][OTf] and [DMEA][Bsa] > [TEA][Bsa]. As triethanolamine had three hydroxyl groups and dimethylethanolamine only one, the regularities of α_*P*_ under the condition of same anion indicated that the number of hydroxyl group had great influence on the value of α_*P*_. The more hydroxyl groups, the smaller α_*P*_. The most likely cause of this phenomenon was that the more hydroxyl groups, the stronger the interactions between molecules, and the smaller the influence of temperature on the distance between molecules, the smaller the coefficient of thermal expansion. The variation of thermal expansion coefficients of investigated PILs were great as they changed from 4.26 ^*^10^−4^ K^−1^ to 5.79 ^*^10^−4^ K^−1^ at temperature (293.15 to 353.15) K. However, the values of PILs were much smaller than the general organic molecular solvents (10^−3^ K^−1^) and greater than the typical molten salt (1–2^*^10^−4^ K^−1^) (Taravillo et al., 2007; Madhusudhan Rao et al., 2013; Khan et al., 2017).

**Table 4:** The thermal expansion coefficient (α_*P*_) of PILs at temperature (293.15 to 353.15) K.

**α × 10^4^ (K^−1^)**										
***T*** **(K)**	**[MDEA][OTf]**	**[DMEA][OTf]**	**[TEA][OTf]**	**[DEA][OTf]**	**[MDEA][mesy]**	**[DEA][mesy]**	**[MDEA][Bsa]**	**[DMEA][Bsa]**	**[TEA][Bsa]**	**[DEA][Bsa]**
293.15	5.35	5.59	4.75	5.30	4.79	4.65	4.70	4.94	4.26	4.70
303.15	5.38	5.62	4.77	5.33	4.82	4.68	4.72	4.96	4.28	4.72
313.15	5.41	5.66	4.80	5.36	4.84	4.70	4.74	4.99	4.30	4.74
323.15	5.44	5.69	4.82	5.39	4.86	4.72	4.76	5.01	4.32	4.76
333.15	5.47	5.72	4.84	5.42	4.89	4.74	4.79	5.04	4.33	4.78
343.15	5.50	5.75	4.87	5.45	4.91	4.76	4.81	5.07	4.35	4.81
353.15	5.53	5.79	4.89	5.48	4.93	4.79	4.83	5.09	4.37	4.83

Standard molar volume of substance stands for the volume of 1 mol pure material at a given temperature and pressure, and it can be calculated according to the following equation (Khan et al., 2017; Nie et al., 2017):

(5) $$\begin{array}{l} V_{m}=\frac{M}{\rho } \end{array}$$

where *V*_*m*_ (cm^3^·mol^−1^) represents the molar volumes of investigated PILs at given temperature and pressure, *M* stands for the molar weight of PILs and ρ represents density of PILs at given temperature and pressure. The molar volumes were calculated and presented in Table 5. The increasing molar volumes of synthesized PILs were noted at temperatures from (293.15 to 353.15) K. Among synthesized PILs, [TEA][Bsa] had the biggest molar volume and [DEA][mesy] had the smallest one. In the same anion, the molar volumes were directly related to cation size, like *V*_m_: [TEA][OTf]>[MDEA][OTf]>[DEA][OTf]≈[DMEA][OTf] and [TEA][Bsa]>[MDEA][Bsa]>[DEA] [Bsa]>[DMEA] [Bsa]. The same change law was applicable to the case of the same cation but different anions. Since the *V*_m_ was largely determined by the molecular spacing, the size of the group had a direct effect on it. The larger the anion and cation, the more obvious the steric effect, the farther they were apart, the larger the *V*_m_. In addition, a similar conclusion could be obtained from the change law of molecular volume, which was defined by the following formula: (Khan et al., 2017)

(6) $$\begin{array}{l} V=\frac{V_{m}}{N_{A}} \end{array}$$

where *V* is the molecular volume of synthesized PILs, *V*_m_ is molar volume calculated by the above formula (5) and *N*_*A*_ is Avogadro's constant equaling to 6.02245 × 10^23^ molecule per mol. The calculated molecular volumes of PILs at temperatures from (293.15 to 353.15) K are presented in Table 5. The change law of molecular volumes corresponded to the change law of molar volumes listed in the same table. Resulting in the conclusion that the size of cations and anions decided the molecular volume. For example, [TEA][Bsa] had the biggest molecular volume among all PILs as it had large benzene ring and three ethanolamine groups. Also, the increase of temperature leaded to the increase of molecular volumes as same as molar volumes because they were relevant to the cation and anion thermal motion. With the increase of temperature, the van der Waal forces of interactions would decrease, which consequently caused an increase in the mobility of the ions (Tariq et al., 2009). Therefore, the higher the temperature, the more intense the thermal motion of the cation and anion, the further they were from each other and the bigger of their molecular volumes.

**Table 5:** Molar volume (*V*_m_) and molecular volume (*V*) of PILs at temperature (293.15 to 353.15) K.

*T* (K)	293.15	303.15	313.15	323.15	333.15	343.15	353.15
**[MDEA][OTf]**							
*V*_m_ (cm^3^·mol^−1^)	184.0	185.0	186.0	187.0	188.0	189.1	190.1
*V* (nm^3^)	0.305	0.307	0.309	0.311	0.312	0.314	0.316
**[DMEA][OTf]**							
*V*_m_ (cm^3^·mol^−1^)	167.3	168.2	169.2	170.2	171.1	172.1	173.1
*V* (nm^3^)	0.278	0.279	0.281	0.283	0.284	0.286	0.287
**[TEA][OTf]**							
*V*_m_ (cm^3^·mol^−1^)	203.4	204.3	205.3	206.3	207.3	208.3	209.3
*V* (nm^3^)	0.338	0.339	0.341	0.343	0.344	0.346	0.348
**[DEA][OTf]**							
*V*_m_ (cm^3^·mol^−1^)	167.8	168.7	169.6	170.5	171.4	172.4	173.3
*V* (nm^3^)	0.279	0.280	0.282	0.283	0.285	0.286	0.288
**[MDEA][mesy]**							
*V*_m_ (cm^3^·mol^−1^)	164.3	165.1	165.9	166.7	167.5	168.3	169.1
*V* (nm^3^)	0.273	0.274	0.275	0.277	0.278	0.279	0.281
**[DEA][mesy])**							
*V*_m_ (cm^3^·mol^−1^)	148.7	149.5	150.2	150.9	151.6	152.3	153.0
*V* (nm^3^)	0.247	0.248	0.249	0.251	0.252	0.253	0.254
**[MDEA][Bsa])**							
*V*_m_ (cm^3^·mol^−1^)	212.4	213.4	214.4	215.5	216.5	217.5	218.5
*V* (nm^3^)	0.353	0.354	0.356	0.358	0.359	0.361	0.363
**[DMEA][Bsa]**							
*V*_m_ (cm^3^·mol^−1^)	194.8	195.8	196.8	197.8	198.8	199.8	200.8
*V* (nm^3^)	0.323	0.325	0.327	0.328	0.330	0.332	0.333
**[TEA][Bsa]**							
*V*_m_ (cm^3^·mol^−1^)	230.9	231.9	232.9	233.9	234.9	236.0	237.0
*V* (nm^3^)	0.383	0.385	0.387	0.388	0.390	0.392	0.393
**[DEA][Bsa]**							
*V*_m_ (cm^3^·mol^−1^)	197.3	198.2	199.2	200.2	201.1	202.1	203.0
*V* (nm^3^)	0.328	0.329	0.331	0.332	0.334	0.336	0.337

### Viscosity (η)

The dynamic viscosity of PILs at the temperatures from (293.15 to 353.15) K are summarized in Table S3. At the investigated temperatures, η: [TEA][Bsa]>[DEA][Bsa]>[MDEA][Bsa]> [DMEA][Bsa]>[DEA][mesy]>[MDEA][mesy]>[DEA][OTf]≈ [TEA][OTf]>[MDEA][OTf] >[DMEA][OTf]. From the order of viscosity of PILs, it was clearly observed that the PILs containing [Bsa] anion had a higher viscosity in the same cation. At the same time, the PILs containing [TEA] cation had maximum viscosity at a given anion in most temperature ranges. However, the viscosity of [TEA][OTf] and [DEA][OTf] was similar at the whole temperature range. As liquid viscosity was caused by the cohesion of molecules, the viscosity change law of investigated PILs could be explained by the interactions of molecules (Yuan et al., 2018). Benzene ring was a large group. Thus, there were strong molecular interactions, specially van der Waals force, between each other. Therefore, PILs including benzene ring had a high viscosity. On the other hand, as [TEA] cation had three hydroxyethyl groups, it could generate strong hydrogen bond between ions, which was thought an important factor affecting the viscosity of ILs (Yang et al., 2017). Hence, PILs containing hydroxyethyl group would have a higher viscosity and their viscosity would increase with the increase of the number of hydroxyethyl groups, as shown in Table S3.

**Figure 3:**
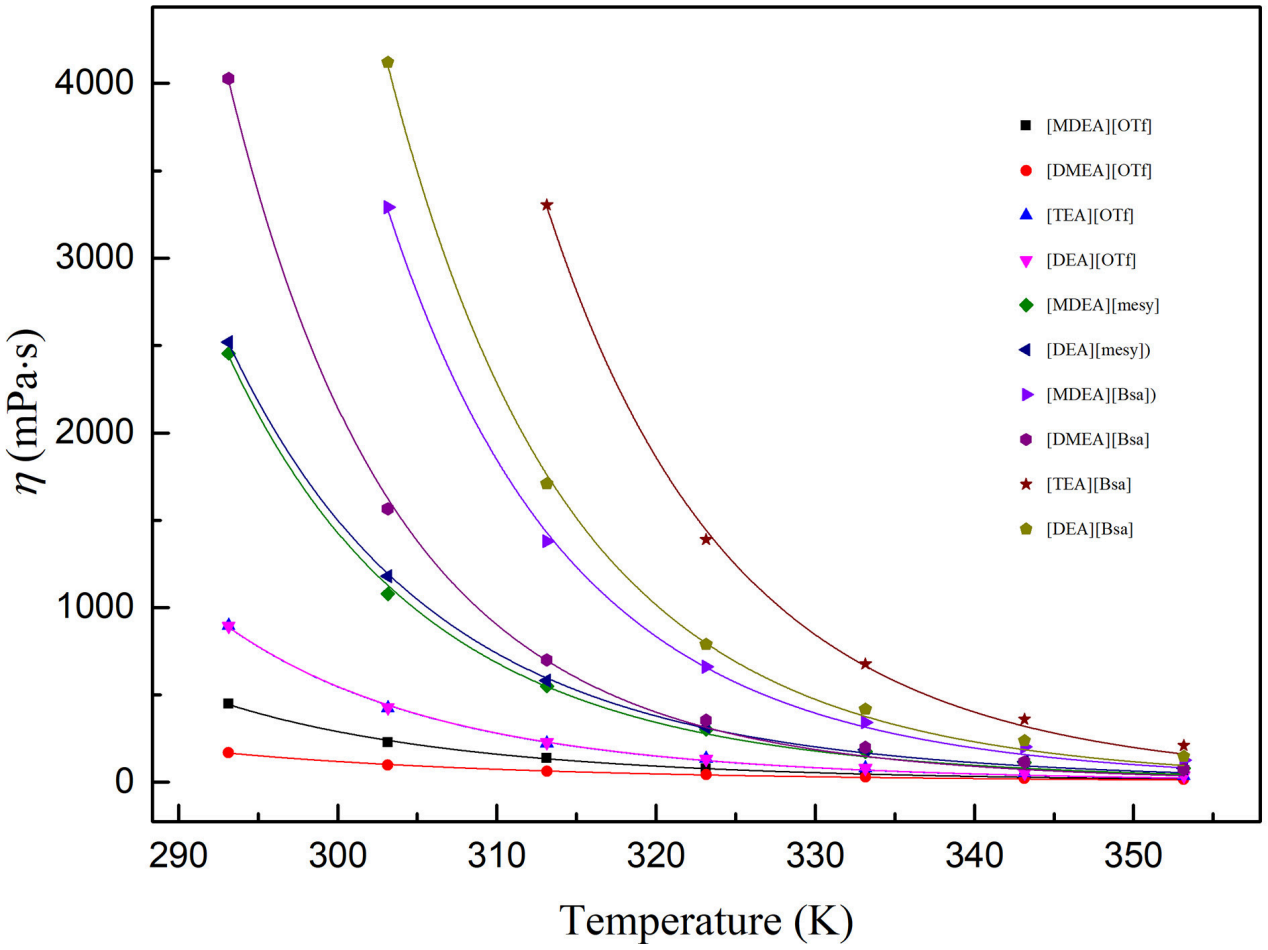
Viscosity of various PIls at temperature (293.15 to 353.15) K. The dots are experimental viscosity data and colored lines are fitting curves according to formula (7).

Arrhenius law was used to the PILs viscosities within the measured temperatures from (293.15 to 353.15) K (Ghatee et al., 2012; Hou et al., 2018; Sayah et al., 2018):

(7) $$\eta =\eta_{0}e^{\left(E_{a,\eta }/RT\right)}$$

where η_0_ is a fitting parameter, *E*_*a*, η_ is the activation energy, R is the universal gas constant (8.314 J·K^−1^·mol^−1^) and *T* is the Kelvin temperature. The parameters are shown in Table 6 and the fitting curves are depicted in Figure 3. From Figure 3, it was obvious that the dynamic viscosity decreased rapidly with the linear increase of temperature and the correlation coefficients (R^2^) indicated the Arrhenius law fitted with experimental data very well. Energy of activation (*E*_*a*, η_) was the least amount of energy required for the ions to move pass through other ones and therefore it could be linked with structure of PILs (Sayah et al., 2018). As seen in Table 6, PILs containing benzene ring had higher *E*_*a*, η_ universally and those containing trifluoromethane sulfonate had lower *E*_*a*, η_, which corresponded with the change law of viscosity. In addition, *E*_*a*, η_ increased with the increase of the number of hydroxyethyl groups when PILs had the same anion. These phenomena could be explained with the alkyl chain size, hydrogen bonding and electrostatic force. When there was a strong hydrogen bonding, electrostatic force or complex structure, it would be more difficult for ions to cross through each other. Thus, high *E*_*a*, η_would be measured.

**Table 6:** The parameter η_0_ and activation energy (*E*_*a*, η_) as well as correlation coefficients (R^2^) of various PILs according to formula (7).

**PILs**	**η_**0**_ (mPa·s)**	***E*_**a, η**_ (kJ·mol^**−1**^)**	***R^**2**^***
[MDEA][OTf]	3.37E-06	45.57	0.9977
[DMEA][OTf]	5.84E-05	36.24	0.9953
[TEA][OTf]	5.69E-07	51.59	0.9978
[DEA][OTf]	5.93E-07	51.49	0.9985
[MDEA][mesy]	1.69E-07	57.01	0.9983
[DEA][mesy])	4.12E-07	54.91	0.9995
[MDEA][Bsa])	1.73E-08	65.45	0.9988
[DMEA][Bsa]	4.87E-09	66.87	0.9989
[TEA][Bsa]	9.10E-09	69.29	0.9985
[DEA][Bsa]	1.25E-08	66.83	0.9990

### Electrical Conductivity (σ)

**Figure 4:**
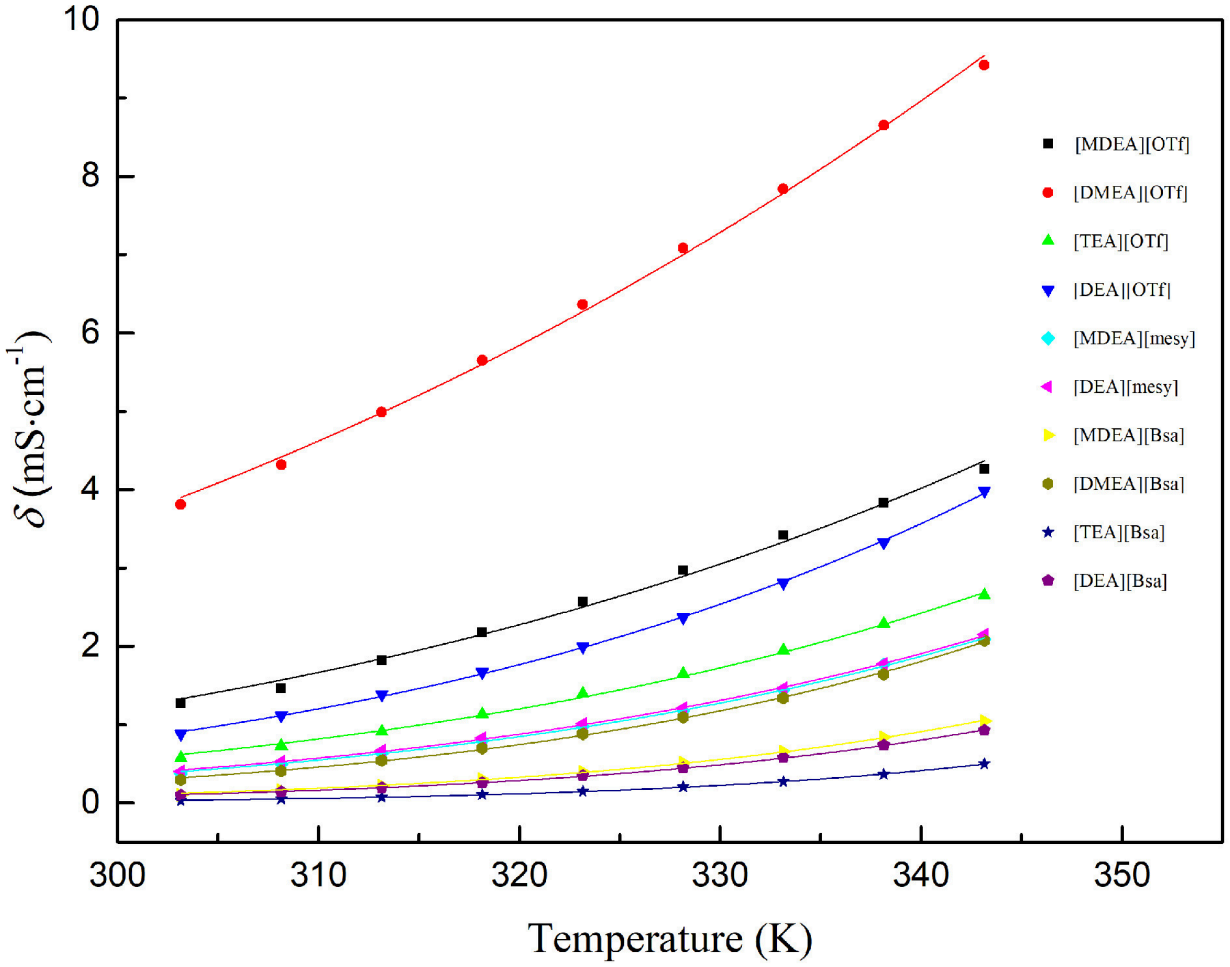
Conductivity of various PILs at temperature (303.15 to 343.15) K. The dots are experimental electrical conductivity data and colored lines are fitting curves according to formula (8).

The electrical conductivity of synthesized PILs are presented in Table S4. Arrhenius law was also used to the PILs electrical conductivity within the measured temperatures from (293.15 to 353.15) K:(Sayah et al., 2018)

(8) $$\sigma =\sigma_{0}e^{\left(-E_{a,\sigma }/RT\right)}$$

where σ_0_ is a fitting parameter, *E*_α, σ_ is the activation energy for the electrical conductivity, R is the universal gas constant (8.314 J·K^−1^·mol^−1^) and *T* is the Kelvin temperature. The parameters are shown in Table 7 and the fitting curves are depicted in Figure 4. As seen in Table 7, it was noted that the correlation coefficients (R^2^) exceeded 0.99. These values indicated that the Arrhenius law fitted very well with the experimental data. As seen in Figure 4, all electrical conductivity of PILs increased with the increase of temperature, this trend fitted with other reports (Sayah et al., 2018; Yuan et al., 2018). In addition, among the synthesized PILs, [DMEA][OTf] had the highest conductivity at all investigated temperatures and [TEA][Bsa] had the lowest one at the same temperature range. Also, the PILs containing anion of benzene ring had lower conductivity and those containing anion of trifluoromethane sulfonate had higher conductivity among investigated PILs, this trend is the opposite of the change law of viscosity discussed in the above section. The calculated *E*_α, σ_ also supports the above discussion. Activation energy for electrical conductivity (*E*_α, σ_) was a parameter to characterize the difficulty level of ions migrating under the electric fields and it was often related to the size or polarity of ions. As seen in Table 7, it was noteworthy that PILs containing benzene ring had the highest *E*_α, σ_, in comparison to others, due to its large structure. Thus, it would be very difficult for this kind of PILs migrating in the liquid (Yuan et al., 2018). What's more, *E*_α, σ_ of PILs containing trifluoromethane sulfonates was low because the molecules were small and polarity was very strong. When PILs had the same anion, the number of hydroxyethyl group was of significance to *E*_α, σ_ and thus to electrical conductivity. For example, the order of *E*_a,σ_: [TEA][OTf] ≈ [DEA][OTf]> [MDEA][OTf]>[DMEA][OTf] and [TEA][Bsa]>[DEA][Bsa]≈[MDEA][Bsa]>[DMEA][Bsa], which indicated that the more hydroxyethyl there is, the more difficult it is for ions to migrate. Because of the ions aggregation/pairing effect, the large ion size could cause the reduction of ion mobility and that could explain the phenomenon described above (Yuan et al., 2018). All activation energy for electrical conductivity of PILs corresponded to conductivity experimental data in the overall trend.

**Table 7:** The fitting parameter σ_0_, activation energy for the electrical conductivity (*E*_α, σ_) and correlation coefficients (R^2^) based on formula (8).

**PILs**	**σ_**0**_ (mS·cm^**−1**^)**	***E_***a*,**_*_σ_ (kJ·mol^**−1**^)**	***R^**2**^***
[MDEA][OTf]	3.56E+04	25.70	0.9940
[DMEA][OTf]	8.42E+03	19.35	0.9979
[TEA][OTf]	1.82E+05	31.73	0.9977
[DEA][OTf]	2.70E+05	31.75	0.9995
[MDEA][mesy]	6.07E+05	35.87	0.9993
[DEA][mesy])	4.64E+05	35.06	0.9994
[MDEA][Bsa])	1.04E+07	45.94	0.9984
[DMEA][Bsa]	2.54E+06	40.01	0.9988
[TEA][Bsa]	1.97E+08	56.48	0.9995
[DEA][Bsa]	1.11E+07	46.47	0.9994

### Dissociation Constants (p*K*a)

Thermodynamic dissociation constants (p*K*a) are used to characterize the extent of dissociation of PILs in an aqueous solution, and the values are good parameters reflecting the degree of relative hydrogen bond donating ability, which are crucial in many chemical processes, like gas absorption and catalysis (Shang et al., 2017).

In general, the acid-base titration curve was used to calculate the p*K*a of explored substance (Xie et al., 2018). In the experiment, the titration curves (pH/V, black dot line) and their first derivative (ΔpH/ΔV, red dot line) curves of all synthesized PILs are shown in Figure S33. The maximum value of the first derivative was used to confirm the volume of titration end point, which was important to the calculation of p*K*a. All calculated p*K*a of investigated PILs are summarized in Table 8. Clearly, various PILs had p*K*a of more than 7.0 and PILs containing [ETA] cation had the highest p*K*a about 9.5 and containing [TEA] had the lowest value about 7.7. Meanwhile, it can be seen that PILs containing the same cation had similar p*K*a, but no apparent relation with anions. This phenomenon could be explained by the mechanism of p*K*a. As the exchangeable proton was on the cation of tertiary amine only, the type of anion did not make sense to p*K*a when its molar concentration was low (0.01 mol/L). Thus, the p*K*a of investigated PILs were related to cation only. However, what deserved our attention was that the number of hydroxyethyl group on tertiary amine was significant in deciding how hard it was for a proton to leave, and thus decide the high or low of p*K*a. Specifically, p*K*a: [ETA]> [DMEA]>[DEA]>[MDEA]>[TEA]. From the regularities of p*K*a, their values decreased with the increase of the number of hydroxyethyl groups. Particularly, PILs containing a [TEA] group had the lowest p*K*a and containing a [ETA] group had the highest p*K*a. In general, the charge distribution of ions was directly related with the interactions between proton and cation, which determined the p*K*a of PILs (Xie et al., 2018). For the series cations of synthesized PILs, the amount of hydroxyethyl groups affected the charge distribution of the cation, which resulted in different p*K*a. This explanation was supported by the p*K*a of PILs containing the same hydroxyethyl groups, such as p*K*a: [DEA]>[MDEA] and [ETA]>[DMEA]. What they have in common was that they had the same number of hydroxyethyl groups. The other group was either methyl or hydrogen, which made them different p*K*a.

**Table 8:** The dissociation constants (p*K*a) of various PILs at 25°C.

Abb.	[MDEA][mesy]	[DMEA][mesy]	[TEA][mesy]	[ETA][mesy]	[DEA][mesy]
p*K*a	8.56	9.23	7.72	9.51	8.79
Abb.	[MDEA][OTf]	[DMEA][OTf]	[TEA][OTf]	[ETA][OTf]	[DEA][OTf]
p*K*a	8.34	9.23	7.73	9.49	8.92
Abb.	[MDEA][Bsa]	[DMEA][Bsa]	[TEA][Bsa]	[ETA][Bsa]	[DEA][Bsa]
p*K*a	8.48	9.17	7.68	9.50	8.79

p*K*a is an important parameter because it indicates the acidity of PILs and has an important reference value in gas absorption (Chowdhury et al., 2013; Hayashi et al., 2014; Furukawa et al., 2017; Shang et al., 2017). As higher p*K*a denotes a stronger hydrogen bond accepting ability of PILs, they can be used for the absorption of acidic gases, like CO_2_. At the same time, lower p*K*a value represents a stronger hydrogen bond donating of PILs and it is conducive to the absorption of basic gases, like NH_3_ (Shang et al., 2017). Therefore, the high p*K*a of synthesized PILs foreshadows their potential applications in acid gas absorption.

### Catalytic Effect for the Conversion of Cyclohexanol

Hydrodeoxygenation (HDO) is an important way to catalyze the conversion of lignin and its derivatives into high-calorific alkane. In this process, deoxygenation is crucial as it makes the whole reaction possible (Chen et al., 2016, 2017; Yang et al., 2019). Brønsted acid (like phosphoric acid), as a deoxidizer, is indispensable for facilitating the deoxygenation reaction in most catalytic systems (Güvenatam et al., 2014; Chen et al., 2016). However, it is noteworthy that acid is harmful to reactors, that inhibits the industrial utilization of lignin. Hence, developing acid free condition for the deoxygenation of lignin and its derivatives is highly desirable.

**Scheme 1:**
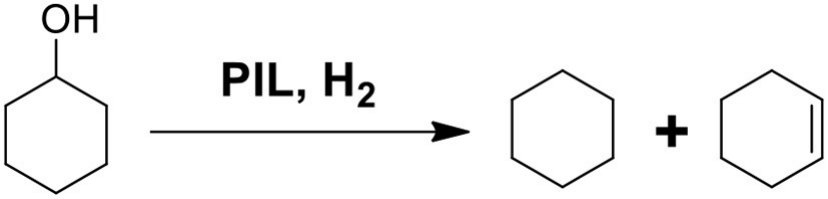
Key steps involved in the formation of cyclohexane or cyclohexene from cyclohexanol.

In order to investigate the potential deoxygenation effect of synthesized PILs, the conversion of cyclohexanol was selected as model reaction as shown in Scheme 1. Table 9 presents catalytic activity of various PILs for deoxidization of cyclohexanol at 120°C for 6 h with the pressure of H_2_ 4 MPa. The GC chromatograms of catalytic results of various PILs are shown in Figures S34–S43. It was noted that all explored PILs showed catalytic activity to a certain extent. Among them, the conversion rate of cyclohexanol in the PILs [MDEA][OTf] and [DEA][OTf] were 100 % and the selectivity of cyclohexane and cyclohexene was 82.1 and 75.5%, respectively. In addition, [DEA][mesy], [MDEA][Bsa], [TEA][Bsa] and [DEA][Bsa] had a conversion rate of cyclohexanol of about 90% in experimental conditions. However, the total selectivity of cyclohexane and cyclohexene of these PILs did go not over 50%, it may be attributed to the intermediate products or products of side-reaction (mainly dimer, cyclohexylcyclohexane) that dissolved in PILs. What's more, the conversation rate of cyclohexanol in [DMEA][OTf], [TEA][OTf], [MDEA][mesy] and [DMEA][Bsa] were 59.8, 74.8, 70.3, and 34.7% with almost <10% selectivity of cyclohexane, the result indicated low catalytic activity for these PILs in the conversion of cyclohexanol. In other reports, it was believed that the catalytic effect of ionic liquids was directly related to the acidity of the PILs (Yan et al., 2010). Therefore, the good catalytic effect of [MDEA][OTf] and [DEA][OTf] may also be attributed to their acidity. In brief, [MDEA][OTf] and [DEA][OTf] showed a promising application in deoxygenation of cyclohexanol during HDO of lignin derived chemicals.

**Table 9:** Catalytic activity of different PILs for the deoxygenation of cyclohexanol.

**PILs**	**Cyclohexane (%)**	**Cyclohexene (%)**	**Conversion (%)**
[MDEA][OTf]	82.1	0	100
[DMEA][OTf]	7.2	0	59.8
[TEA][OTf]	3.9	0	74.8
[DEA][OTf]	0	75.5	100
[MDEA][mesy]	6.2	0	70.3
[DEA][mesy]	41.8	0	89.4
[MDEA][Bsa]	8.1	21.2	91.8
[DMEA][Bsa]	7.1	25.8	34.7
[TEA][Bsa]	11.2	32.1	97.8
[DEA][Bsa]	4.7	38.9	95.2

*Reaction conditions: phenol, 1 mmol; ionic liquid, 2.0 g; H_2_ pressure, 4 MPa; reaction temperature, 120°C (referred to the constant-temperature air bath temperature); reaction time, 6 h. The conversion and yields were determined by GC using n-dodecane as an internal standard*.

## Conclusion

A series of 2-hydroxyethylammonium sulfonate-based PILs were synthesized as well as characterized via NMR, FT-IR and elemental analysis. The thermodynamics methods of TGA and DAS were used to determine *T*_m_ and *T*_d_ of synthesized PILs, results showed that most of PILs had *T*_m_ below 0°C. Also, the range of *T*_d_ for the synthesized PILs was wide, the value was mainly determined by cations and barely affected by anions. Meanwhile, the physicochemical properties, like density, viscosity and electrical conductivity, were also explored. The results indicated that these physicochemical properties were related to their structure tightly and the interactions between anions and cations played a crucial role in determining them. In addition, the thermodynamic dissociation constants of synthesized PILs confirmed that the values showed a strong relation with their cations' structure. Specifically, the regularities of p*K*a manifested that the more hydroxyethyl groups in cation, the lower the p*K*a for PILs. In general, the values of p*K*a of various synthesized PILs exceeded 7.0, which indicated a potential application in the field of acidic gases absorption. What's more, [MDEA][OTf] and [DEA][OTf] showed efficient catalytic activity in the reaction of deoxidation with cyclohexanol as the substrate at certain conditions, the conversion rate of cyclohexanol reached up to 100 % and the selectivity of cyclohexane and cyclohexene was 82.1 and 75.5%, respectively. The results indicated that these PILs had a great potential in the application of deoxygenation reaction during HDO processes.

## Author Contributions

GC designed the research. GC and LL prepared the samples and did determinations. GC and SY were involved in catalytic reactions. GC, SY, QZ, XL, JX, and SZ wrote the manuscript.

### Conflict of Interest Statement

The authors declare that the research was conducted in the absence of any commercial or financial relationships that could be construed as a potential conflict of interest.
