# Supplementary material for: Physicochemical Properties of Various 2-Hydroxyethylammonium Sulfonate -Based Protic Ionic Liquids and Their Potential Application in Hydrodeoxygenation
Source: Front Chem. 2019 Apr 5;7:196. doi: 10.3389/fchem.2019.00196 (PMC6460099; doi:10.3389/fchem.2019.00196)
Supplement: Supplementary file 1 [file Data_Sheet_1.docx]

**Physicochemical Properties of Various 2-Hydroxyethylammonium Sulfonate -Based Protic Ionic Liquids and Their Potential Application in Hydrodeoxygenation**

**Supplementary Material**

1. ^1^H-NMR and ^13^C-NMR spectra of the synthesized PILs in DMSO-*d*_6_.

(a)

6

1

4

3

5

2

(b)

2

3

1

4

**Figure S1 |** ^1^H-NMR (a) and ^13^C-NMR (b) spectrum of [MDEA][mesy] in DMSO-*d*_6_.

^1^H NMR (600 MHz, DMSO-*d*_6_, TMS): δ = 9.11 (s, 1H), 5.27 (t, *J* = 4.9 Hz, 2H), 3.74 (q, *J* = 4.9 Hz, 4H), 3.21 (t, *J* = 5.4 Hz, 4H), 2.82 (s, 3H), 2.34 (s, 3H).

^13^C NMR (151 MHz, DMSO-*d_6_*): δ = 57.64, 55.66, 40.93, 40.17.

(a)

6

1

4

3

5

2

(b)

2

3

1

4

**Figure S2 |** ^1^H-NMR (a) and ^13^C-NMR (b) spectrum of [DMEA][mesy] in DMSO-*d*_6_.

^1^H NMR (600 MHz, DMSO-*d*_6_, TMS): δ = 9.26 (s, 1H), 5.33 (t, *J* = 4.8 Hz, 1H), 3.70 (m, 2H), 3.15(m, 2H), 2.79 (s, 6H), 2.34 (s, 3H).

^13^C NMR (151 MHz, DMSO-*d_6_*): δ = 58.77, 55.56, 43.03, 40.18.

(a)

5

1

4

2

3

(b)

1

2

3

**Figure S3 |** ^1^H-NMR (a) and ^13^C-NMR (b) spectrum of [TEA][mesy] in DMSO-*d*_6_.

^1^H NMR (600 MHz, DMSO-*d*_6_, TMS): δ = 8.78 (s,1H), 5.27 (s, 3H), 3.75 (t, *J* = 5.4 Hz, 6H), 3.31 (q, *J* = 5.0 Hz, 6H), 2.34 (s, 3H).

^13^C NMR (151 MHz, DMSO-*d*_6_): δ = 55.61, 55.57, 40.17.

(a)

5

3

2

4

1

(b)

1

2

3

**Figure S4 |** ^1^H-NMR (a) and ^13^C-NMR (b) spectrum of [ETA][mesy] in DMSO-*d*_6_.

^1^H NMR (600 MHz, DMSO-*d*_6_, TMS): δ = 7.74 (s, 3H), 5.13 (t, *J* = 5.1 Hz, 1H), 3.58 (q, *J* = 4.7 Hz, 2H), 2.86 (m, 2H), 2.36 (s, 3H).

^13^C NMR (151 MHz, DMSO-*d*_6_): δ = 57.90, 41.65, 40.17.

(a)

5

3

2

1

(b)

1

2

3

**Figure S5 |** ^1^H-NMR (a) and ^13^C-NMR (b) spectrum of [DEA][mesy] in DMSO-*d*_6_.

^1^H NMR (600 MHz, DMSO-*d*_6_, TMS): δ = 8.38 (s, 2H), 3.66 (t, *J* = 5.4 Hz, 5H), 3.02 (m, *J* = 5.7 Hz, 4H), 2.36 (s, 3H).

^13^C NMR (151 MHz, DMSO-*d*_6_): δ = 56.74, 49.30, 40.18.

(a)

5

2

4

3

1

(b)

2

3

1

4

**Figure S6 |** ^1^H-NMR (a) and ^13^C-NMR (b) spectrum of [MDEA][OTf] in DMSO-*d*_6_.

^1^H NMR (600 MHz, DMSO-*d*_6_, TMS): δ = 9.07 (s, 1H), 5.26 (s, 2H), 3.73 (m, *J* = 5.3 Hz, 4H), 3.27 (m, 2H), 3.13 (m, 2H), 2.81 (d, *J* = 4.7 Hz, 3H).

^13^C NMR (151 MHz, DMSO-*d*_6_): δ = 122.22, 57.61, 55.58, 40.85.

(a)

1

2

5

4

3

(b)

4

2

3

1

**Figure S7 |** ^1^H-NMR (a) and ^13^C-NMR (b) spectrum of [DMEA][OTf] in DMSO-*d*_6_.

^1^H NMR (600 MHz, DMSO-*d*_6_, TMS): δ = 9.20 (s, 1H), 5.32 (t, *J* = 4.7 Hz, 1H), 3.69 (m, *J* = 5.0 Hz, 2H), 3.13 (m, *J* = 4.8 Hz, 2H), 2.77 (s, 6H).

^13^C NMR (151 MHz, DMSO-*d*_6_): δ = 122.21, 58.77, 55.47, 43.00.

(a)

1

4

3

2

(b)

3

1/2

**Figure S8 |** ^1^H-NMR (a) and ^13^C-NMR (b) spectrum of [TEA][OTf] in DMSO-*d*_6_.

^1^H NMR (600 MHz, DMSO-*d*_6_, TMS): δ = 8.76 (s, 1H), 5.25 (t, *J* = 4.9 Hz, 3H), 3.75 (q, *J* = 5.1 Hz, 6H), 3.29 (t, *J* = 5.4 Hz, 6H).

^13^C NMR (151 MHz, DMSO-*d*_6_): δ =120.07, 55.60.

(a)

3

2

4

1

(b)

3

1

2

**Figure S9 |** ^1^H-NMR (a) and ^13^C-NMR (b) spectrum of [ETA][OTf] in DMSO-*d*_6_.

^1^H NMR (600 MHz, DMSO-*d*_6_, TMS): δ = 7.67 (s, 3H), 5.13 (s, 1H), 3.57 (t, *J* = 5.1 Hz, 2H), 2.86 (m, *J* = 5.4 Hz, 2H).

^13^C NMR (151 MHz, DMSO-*d*_6_): δ = 122.21, 57.89, 41.63.

(a)

3

2

4

1

(b)

3

1

2

**Figure S10 |** ^1^H-NMR (a) and ^13^C-NMR (b) spectrum of [DEA][OTf] in DMSO-*d*_6_.

^1^H NMR (600 MHz, DMSO-*d*_6_, TMS): δ = 8.35 (s, 2H), 5.18 (s, 2H), 3.65 (t, *J* = 5.1 Hz, 4H), 3.01 (m, *J* = 5.4 Hz, 4H).

^13^C NMR (151 MHz, DMSO-*d*_6_): δ = 122.21, 56.70, 49.26.

(a)

1

4

3

7/8

6

2

5

(b)

4

7

6

5

2

3

1

**Figure S11 |** ^1^H-NMR (a) and ^13^C-NMR (b) spectrum of [MDEA][Bsa] in DMSO-*d*_6_.

^1^H NMR (600 MHz, DMSO-*d*_6_, TMS): δ = 9.07 (s, 1H), 7.83 – 7.48 (m, 2H), 7.46 – 7.15 (m, 3H), 4.95 (s, 2H), 3.73 (t, 4H), 3.34 – 3.22 (m, 2H), 3.14 (m, 2H), 2.81 (d, *J* = 4.9 Hz, 3H).

1

2

3

4

5

6

7/8

^13^C NMR (151 MHz, DMSO-*d*_6_): δ = 128.90, 128.12, 125.94, 57.60, 55.61, 40.87.

(a)

2

6

7/8

3

4

5

1

(b)

4

7

6

5

2

3

1

**Figure S12 |** ^1^H-NMR (a) and ^13^C-NMR (b) spectrum of [DMEA][Bsa] in DMSO-*d*_6_.

^1^H NMR (600 MHz, DMSO-*d*_6_, TMS): δ = 9.20 (s, 1H), 7.72 – 7.53 (m, 2H), 7.42 – 7.22 (m, 3H), 5.17 (s, 1H), 3.68 (t, 2H), 3.14 (q, *J* = 5.3 Hz, 2H), 2.78 (d, *J* = 4.7 Hz, 6H).

1

3

2

4

6/7

5

^13^C NMR (151 MHz, DMSO-*d*_6_): δ = 148.58, 129.01, 128.18, 125.94, 58.75, 55.56, 43.02.

(a)

1

5

6/7

4

2

3

(b)

3

6

5

4

1

2

**Figure S13 |** ^1^H-NMR (a) and ^13^C-NMR (b) spectrum of [TEA][Bsa] in DMSO-*d*_6_.

^1^H NMR (600 MHz, DMSO-*d*_6_, TMS): δ = 8.75 (s, 1H), 7.71 – 7.49 (m, 2H), 7.43 – 7.17 (m, 3H), 5.25 (s, 3H), 3.75 (t, *J* = 5.3 Hz, 6H), 3.30 (q, 6H).

^13^C NMR (151 MHz, DMSO-*d*_6_): δ = 148.73, 148.70, 128.93, 128.90, 128.13, 125.94, 55.62, 55.57.

(a)

1

2

3

4

5

6/7

(b)

3

6

5

4

1

2

**Figure S14 |** ^1^H-NMR (a) and ^13^C-NMR (b) spectrum of [ETA][Bsa] in DMSO-*d*_6_.

^1^H NMR (600 MHz, DMSO-*d*_6_, TMS): δ = 7.71 (s, 3H), 7.64 – 7.57 (m, 2H), 7.42 – 7.20 (m, 3H), 5.13 (t, *J* = 5.0 Hz, 1H), 3.57 (m, 2H), 2.86 (t, 2H).

3

2

4

6/7

5

1

^13^C NMR (151 MHz, DMSO-*d*_6_): δ = 148.53, 129.01, 128.17, 125.93, 57.89, 41.65.

(a)

1

5

6/7

4

2

3

(b)

3

6

5

4

1

2

**Figure S15 |** ^1^H-NMR (a) and ^13^C-NMR (b) spectrum of [DEA][Bsa] in DMSO-*d*_6_.

^1^H NMR (600 MHz, DMSO-*d*_6_, TMS): δ = 8.36 (s, 2H), 7.67 – 7.55 (m, 2H), 7.40 – 7.22 (m, 3H), 5.02 (s, 1H), 3.65 (t, 5H), 3.01 (m, *J* = 5.6 Hz, 4H).

^13^C NMR (151 MHz, DMSO-*d*_6_): δ = 128.92, 128.13, 125.94, 56.72, 49.27.

1. FT-IR spectra of explored PILs.

**Figure S16 |** FT-IR spectrum of [MDEA][mesy].

FT-IR (3353 cm^-1^, -OH; 1460-1470 cm^-1^, -CH_3_; 1210-1180 cm^-1^, C-O and C-S; 1056 cm^-1^, C-N; 783 cm^-1^, CH_2_-CH_2_; 560-520 cm^-1^, Sulfonic acid group).

**Figure S17 |** FT-IR spectrum of [DMEA][mesy].

FT-IR (3371 cm^-1^, -OH; 1469cm^-1^, -CH_3_; 1210-1180 cm^-1^, C-O and C-S; 1057cm^-1^, C-N; 780 cm^-1^, CH_2_-CH_2_; 560-520 cm^-1^, Sulfonic acid group).

**Figure S18 |** FT-IR spectrum of [TEA][mesy].

FT-IR (3357 and 1404 cm^-1^, -OH; 1210-1180 cm^-1^, C-O and C-S; 1059 cm^-1^, C-N; 783 cm^-1^, CH_2_-CH_2_; 560-520 cm^-1^, Sulfonic acid group).

**Figure S19 |** FT-IR spectrum of [ETA][mesy].

FT-IR (3372 cm^-1^, -OH; 3006 cm-1, N-H; 1210-1180 cm^-1^, C-O and C-S; 1057 cm^-1^, C-N; 783 cm^-1^, CH_2_-CH_2_; 560-520 cm^-1^, Sulfonic acid group).

**Figure S20 |** FT-IR spectrum of [DEA][mesy].

FT-IR (3371 cm^-1^, -OH; 3100-2900 cm^-1^, N-H; 1210-1180 cm^-1^, C-O and C-S; 1057 cm^-1^, C-N; 783 cm^-1^, CH_2_-CH_2_; 560-520 cm^-1^, Sulfonic acid group).

**Figure S21 |** FT-IR spectrum of [MDEA][OTf].

FT-IR (3442 cm^-1^, -OH; 1471 cm^-1^, -CH_3_; 1280-1200 cm^-1^, C-O and C-S; 1631, 1167 and 642 cm^-1^, -CF_3_; 1032 cm^-1^, C-N; 762 cm^-1^, CH_2_-CH_2_; 560-510 cm^-1^, Sulfonic acid group).

**Figure S22 |** FT-IR spectrum of [DMEA][OTf].

FT-IR (3465 cm^-1^, -OH; 1475 cm^-1^, -CH_3_; 1280-1200 cm^-1^, C-O and C-S; 1630, 1169 and 638 cm^-1^, -CF_3_; 1031 cm^-1^, C-N; 765-760 cm^-1^, CH_2_-CH_2_; 560-510 cm^-1^, Sulfonic acid group).

**Figure S23 |** FT-IR spectrum of [TEA][OTf].

FT-IR (3356 cm^-1^, -OH; 1280-1200 cm^-1^, C-O and C-S; 1174 and 642 cm^-1^, -CF_3_; 1028 cm^-1^, C-N; 765-760 cm^-1^, CH_2_-CH_2_; 560-510 cm^-1^, Sulfonic acid group).

**Figure S24 |** FT-IR spectrum of [ETA][OTf].

FT-IR (3446 cm^-1^, -OH; 3145 cm^-1^, N-H; 1406 cm^-1^, -CH_3_; 1280-1200 cm^-1^, C-O and C-S; 1612, 1174 and 642 cm^-1^, -CF_3_; 1032 cm^-1^, C-N; 765-760 cm^-1^, CH_2_-CH_2_; 560-510 cm^-1^, Sulfonic acid group).

**Figure S25 |** FT-IR spectrum of [DEA][OTf].

FT-IR (3442 cm^-1^, -OH; 3089 cm^-1^, N-H; 1454 cm^-1^, -CH_3_; 1280-1200 cm^-1^, C-O and C-S; 1603, 1169 and 642 cm^-1^, -CF_3_; 1032 cm^-1^, C-N; 765-760 cm^-1^, CH_2_-CH_2_; 560-510 cm^-1^, Sulfonic acid group).

**Figure S26 |** FT-IR spectrum of [MDEA][Bsa].

FT-IR (3375 cm^-1^, -OH; 1444 cm^-1^, -CH_3_; 1280-1180 cm^-1^, C-O and C-S; 780-650 cm^-1^, Benzene ring; 615 and 565 cm^-1^, Sulfonic acid group).

**Figure S27** | FT-IR spectrum of [DMEA][Bsa].

FT-IR (3398 cm^-1^, -OH; 1446 cm^-1^, -CH_3_; 1280-1180 cm^-1^, C-O and C-S; 780-650 cm^-1^, Benzene ring; 611 and 567 cm^-1^, Sulfonic acid group).

**Figure S28 |** FT-IR spectrum of [TEA][Bsa].

FT-IR (3351 cm^-1^, -OH; 1280-1180 cm^-1^, C-O and C-S; 780-650 cm^-1^, Benzene ring; 550-600 cm^-1^, Sulfonic acid group).

**Figure S29 |** FT-IR spectrum of [ETA][Bsa].

FT-IR (3394 cm^-1^, -OH; 3055 cm^-1^, N-H; 1446 cm^-1^, -CH_2_CH_2_-; 1280-1180 cm^-1^, C-O and C-S; 780-650 cm^-1^, Benzene ring; 615 and 569 cm^-1^, Sulfonic acid group).

**Figure S30 |** FT-IR spectrum of [DEA][Bsa].

FT-IR (3384 cm^-1^, -OH; 3055 cm^-1^, N-H; 1446 cm^-1^, -CH_2_CH_2_-; 1280-1180 cm^-1^, C-O and C-S; 780-650 cm^-1^, Benzene ring; 613 and 565 cm^-1^, Sulfonic acid group).

**Table S1 |** The water content of synthesized PILs measured by Karl Fischer titrator

| Abb. | [MDEA][mesy] | [DMEA][mesy] | [TEA][mesy] | [ETA][mesy] | [DEA][mesy] |
| --- | --- | --- | --- | --- | --- |
| Water content  (ppm) | 1045 | 1465 | 1988 | 1534 | 1601 |
| Abb. | [MDEA][OTf] | [DMEA][OTf] | [TEA][OTf] | [ETA][OTf] | [DEA][OTf] |
| Water content (ppm) | 2297 | 2457 | 2912 | 3415 | 3047 |
| Abb. | [MDEA][Bsa] | [DMEA][Bsa] | [TEA][Bsa] | [ETA][Bsa] | [DEA][Bsa] |
| Water content (ppm) | 1834 | 1627 | 2198 | 1978 | 1632 |

1. Elemental analysis (CHNS) results of synthesized PILs

N-methyldiethanolamine methanesulfonate [MDEA][mesy]

CHNS analysis: C_6_H_17_N_1_S_1_O_5_, Mol. Wt.: 215.27

Calculated: C, 33.44; H, 7.90; N, 6.50; S, 14.90

Found: C, 33.55; H, 8.03; N, 6.67; S, 14.89

N, N-dimethylethanolamine methanesulfonate [DMEA][mesy]

CHNS analysis: C_5_H_15_N_1_S_1_O_4_, Mol. Wt.: 185.25

Calculated; C, 32.39; H, 8.10; N, 7.56; S, 17.27

Found: C, 32.31; H, 8.49; N, 7.82; S, 17.42

Triethanolamine methanesulfonate [TEA][mesy]

CHNS analysis: C_7_H_19_N_1_S_1_O_6_, Mol. Wt.: 245.3

Calculated; C, 34.24; H, 7.75; N, 5.71; S, 13.05

Found: C, 34.51; H, 8.10; N, 6.03; S, 13.10

Ethanolamine methanesulfonate [ETA][mesy]

CHNS analysis: C_3_H_11_N_1_S_1_O_4_, Mol. Wt.: 157.19

Calculated; C, 22.90; H, 7.00; N, 8.91; S, 20.36

Found: C, 23.03; H, 7.27; N, 9.03; S, 20.75

Diethanolamine methanesulfonate [DEA][mesy]

CHNS analysis: C_5_H_15_N_1_S_1_O_5_, Mol. Wt.: 201.25

Calculated; C, 29.81; H, 7.45; N, 6.96; S, 15.90

Found: C, 29.45; H, 7.76; N, 7.11; S, 15.94

N-methyldiethanolamine trifluoromethanesulfonate [MDEA][OTf]

CHNS analysis: C_6_H_14_N_1_S_1_O_5_F_3_, Mol. Wt.: 269.23

Calculated; C, 26.74; H, 5.20; N, 5.20; S, 11.89

Found: C, 26.83; H, 5.55; N, 5.58; S, 12.26

N, N-dimethylethanolamine trifluoromethanesulfonate [DMEA][OTf]

CHNS analysis: C_5_H_12_N_1_S_1_O_4_F_3_, Mol. Wt.: 239.21

Calculated; C, 25.08; H, 5.02; N, 5.85; S, 13.38

Found: C, 25.25; H, 5.42; N, 6.19; S, 13.31

Triethanolamine trifluoromethanesulfonate [TEA][OTf]

CHNS analysis: C_7_H_16_N_1_S_1_O_6_F_3_, Mol. Wt.: 299.26

Calculated; C, 28.07; H, 5.35; N, 4.68; S, 10.69

Found: C, 28.42; H, 5.55; N, 4.82; S, 10.76

Ethanolamine trifluoromethanesulfonate [ETA][OTf]

CHNS analysis: C_3_H_8_N_1_S_1_O_4_F_3_, Mol. Wt.: 211.15

Calculated; C, 17.05; H, 3.79; N, 6.63; S, 15.16

Found: C, 17.23; H, 3.72; N, 6.98; S, 15.52

Diethanolamine trifluoromethanesulfonate [DEA][OTf]

CHNS analysis: C_5_H_12_N_1_S_1_O_5_F_3_, Mol. Wt.: 255.21

Calculated; C, 23.51; H, 4.70; N, 5.49; S, 12.54

Found: C, 23.53; H, 4.81; N, 5.62; S, 12.78

N-methyldiethanolamine benzenesulfonate [MDEA][Bsa]

CHNS analysis: C_11_H_19_N_1_S_1_O_5_, Mol. Wt.: 277.34

Calculated; C, 47.60; H, 6.85; N, 5.05; S, 11.54

Found: C, 47.69; H, 6.98; N, 5.25; S, 11.69

N, N-dimethylethanolamine benzenesulfonate [DMEA][Bsa]

CHNS analysis: C_10_H_17_N_1_S_1_O_4_, Mol. Wt.: 247.32

Calculated; C, 48.52; H, 6.87; N, 5.66; S, 12.94

Found: C, 48.50; H, 6.45; N, 5.25; S, 12.73

Triethanolamine benzenesulfonate [TEA][Bsa]

CHNS analysis: C_12_H_21_N_1_S_1_O_6_, Mol. Wt.: 307.37

Calculated; C, 46.85; H, 6.83; N, 4.55; S, 10.41

Found: C, 47.28; H, 7.20; N, 4.73; S, 10.16

Ethanolamine benzenesulfonate [ETA][Bsa]

CHNS analysis: C_8_H_13_N_1_S_1_O_4_, Mol. Wt.: 219.26

Calculated; C, 43.78; H, 5.93; N, 6.39; S, 14.59

Found: C, 44.20; H, 6.04; N, 6.36; S, 15.03

Diethanolamine benzenesulfonate [DEA][Bsa]

CHNS analysis: C_10_H_17_N_1_S_1_O_5_, Mol. Wt.: 263.32

Calculated; C, 45.57; H, 6.46; N, 5.32; S, 12.15

Found: C, 45.98; H, 6.96; N, 5.75; S, 12.37

1. The TGA curves of synthesized PILs (black line) and the determination line of 5 % weight loss of samples (red dot line).

**Figure S31 |** The TGA curves (black line) of synthesized PILs from room temperature to 800 ℃ and decomposition temperature determination line (red dot line).

1. The DSC curves of synthesized PILs at temperature (-50 to 350) ℃.

**Figure S32 |** The DSC curves (black line) of synthesized PILs from (-50 to 350) ℃.

**Table S2 |** Density data of synthesized PILs at temperature (293.15 to 353.15) K.

| *ρ* (g∙cm^-1^) | | | | | | | | | | | |
| --- | --- | --- | --- | --- | --- | --- | --- | --- | --- | --- | --- |
| *T* (K) | [MDEA][OTf] | [DMEA][OTf] | [TEA][OTf] | [DEA][OTf] | [MDEA][mesy] | [DEA][mesy]) | [MDEA][Bsa]) | [DMEA][Bsa] | [TEA][Bsa] | | [DEA][Bsa] |
| 293.15 | 1.4634 | 1.4300 | 1.4715 | 1.5211 | 1.3105 | 1.3530 | 1.3059 | 1.2696 | 1.3311 | 1.3347 | |
| 303.15 | 1.4554 | 1.4218 | 1.4645 | 1.5129 | 1.3040 | 1.3466 | 1.2996 | 1.2631 | 1.3254 | 1.3282 | |
| 313.15 | 1.4474 | 1.4137 | 1.4575 | 1.5047 | 1.2976 | 1.3401 | 1.2934 | 1.2567 | 1.3197 | 1.3219 | |
| 323.15 | 1.4396 | 1.4057 | 1.4505 | 1.4966 | 1.2913 | 1.3338 | 1.2872 | 1.2505 | 1.3140 | 1.3155 | |
| 333.15 | 1.4318 | 1.3977 | 1.4435 | 1.4886 | 1.2851 | 1.3276 | 1.2811 | 1.2443 | 1.3083 | 1.3093 | |
| 343.15 | 1.4241 | 1.3898 | 1.4365 | 1.4806 | 1.2789 | 1.3214 | 1.2751 | 1.2381 | 1.3027 | 1.3032 | |
| 353.15 | 1.4164 | 1.3820 | 1.4296 | 1.4727 | 1.2728 | 1.3152 | 1.2691 | 1.2319 | 1.2971 | 1.2971 | |

**Table S3 |** Viscosity data of synthesized PILs at temperature (293.15 to 353.15) K.

| *η* (mPa∙s) | | | | | | | | | | | |
| --- | --- | --- | --- | --- | --- | --- | --- | --- | --- | --- | --- |
| *T* (K) | [MDEA][OTf] | [DMEA][OTf] | [TEA][OTf] | | [DEA][OTf] | [MDEA][mesy] | [DEA][mesy]) | [MDEA][Bsa]) | [DMEA][Bsa] | [TEA][Bsa] | [DEA][Bsa] |
| 293.15 | 449.0 | 170.2 | 894.8 | 894.1 | | 2455 | 2519 |  | 4027 |  |  |
| 303.15 | 228.8 | 97.18 | 422.7 | 425.6 | | 1078 | 1180 | 3291 | 1567 |  | 4120 |
| 313.15 | 136.7 | 62.59 | 223.3 | 227.6 | | 549.5 | 581.5 | 1381 | 700.7 | 3305 | 1709 |
| 323.15 | 77.82 | 42.55 | 136.1 | 132.2 | | 301.7 | 315.1 | 660.9 | 354.7 | 1390 | 789.3 |
| 333.15 | 51.36 | 30.28 | 81.38 | 79.70 | | 175.4 | 187.9 | 342.6 | 198.9 | 676.9 | 417.6 |
| 343.15 | 35.65 | 22.41 | 53.90 | 52.49 | | 112.6 | 119.2 | 202.1 | 116.7 | 359.8 | 237.8 |
| 353.15 | 26.01 | 17.13 | 37.61 | 36.23 | | 80.00 | 80.00 | 125.8 | 75.68 | 209.6 | 146.6 |

**Table S4 |** Electrical conductivity data of synthesized PILs at temperature (303.15 to 343.15) K.

| *σ* (mS·cm^-1^) | | | | | | | | | | | | |
| --- | --- | --- | --- | --- | --- | --- | --- | --- | --- | --- | --- | --- |
| *T* (K) | [MDEA][OTf] | [DMEA][OTf] | [TEA][OTf] | | [DEA][OTf] | [MDEA][mesy] | [DEA][mesy]) | [MDEA][Bsa]) | [DMEA][Bsa] | [TEA][Bsa] | | [DEA][Bsa] |
| 303.15 | 1.27 | 3.81 | 0.572 | 0.884 | | 0.377 | 0.405 | 0.101 | 0.296 | 0.032 | 0.098 | |
| 308.15 | 1.46 | 4.31 | 0.729 | 1.12 | | 0.497 | 0.526 | 0.160 | 0.408 | 0.050 | 0.139 | |
| 313.15 | 1.82 | 4.99 | 0.910 | 1.38 | | 0.636 | 0.670 | 0.223 | 0.544 | 0.075 | 0.194 | |
| 318.15 | 2.18 | 5.65 | 1.13 | 1.67 | | 0.796 | 0.830 | 0.303 | 0.701 | 0.108 | 0.261 | |
| 323.15 | 2.57 | 6.36 | 1.40 | 2.00 | | 0.978 | 1.01 | 0.399 | 0.883 | 0.150 | 0.347 | |
| 328.15 | 2.97 | 7.09 | 1.65 | 2.37 | | 1.18 | 1.21 | 0.515 | 1.10 | 0.204 | 0.450 | |
| 333.15 | 3.42 | 7.84 | 1.95 | 2.81 | | 1.44 | 1.46 | 0.660 | 1.34 | 0.273 | 0.577 | |
| 338.15 | 3.83 | 8.65 | 2.29 | 3.33 | | 1.77 | 1.78 | 0.841 | 1.65 | 0.366 | 0.740 | |
| 343.15 | 4.26 | 9.42 | 2.65 | 3.98 | | 2.09 | 2.15 | 1.05 | 2.07 | 0.500 | 0.928 | |

1. Determination of dissociation constants p*K*a

The specific operation steps of determination of dissociation constants p*K*a and derivation procedures are displayed in the following:

1.Take a certain amount of ultra-pure water (provide by Milli-Q direct 8) and boil it to remove carbon dioxide. Then the water was used to prepare 0.1mol/L KCl solution, which was used to configurate the following aqueous samples.

2. Using 250 ml volumetric flask and 0.1mol/L KCl solution to prepare 0.01 mol/L NaOH and PILs solutions, respectively. Then weighing the dry potassium hydrogen phthalate (KHP) to prepare KHP aqueous solution with molar concentration of 0.01 mol/L as standard solution, and it was used to titrate NaOH solution with this solution in order to get its exact molar concentration (temperature 25 ± 0.1 ℃, similarly hereinafter).

3. 15 mL PILs solution was accurately removed by pipette and placed in a self-made water jacketed glassware. Stirring the N_2_ protected PILs solution by magneton in glassware. The pH value of the solution was measured by Ag–AgCl/glass combination electrode on a Mettler Toledo pH meter (three standard buﬀer solutions with the pH value of 4.00, 6.86 and 9.18 were used to calibrate the electrode before measurement). Then the calibrated NaOH solution was added to the glassware drop by drop. When pH meter reading was stabilized, the reading was recorded. Therefore, the relationship between the volume of NaOH and the pH of the solution was obtained.

Dissociation equilibrium of PILs can be expressed as the following process:

$[\mathrm{RH}^{+}$][$\mathrm{MSO}_{3}^{-}$] + SH ⇌ $\left[ \left[ R^{-} \right]^{+} \right]\left[ \mathrm{MS}O_{3}^{-} \right]$ + $\left[ SH_{2}^{+} \right]$ (S1)

where SH stands for a solvent that can take a proton, $SH_{2}^{+}$ denotes a solvated proton, R and M stand for a particular group in cation and anion of synthesized PILs, respectively.$[\mathrm{RH}^{+}$] and $\left[ R^{-} \right]^{+}$stand for the concentration of acid cation and the conjugate base of the cation of the PILs, respectively. according to the above equilibrium, the dissociation constant can be expressed by the following equation:

$K_{a}=\frac{\left[ R^{-} \right]^{+}*\left[ \mathrm{MS}O_{3}^{-} \right]*\left[ \mathrm{SH}_{2}^{+} \right]}{\left[ \mathrm{RH}^{+} \right]*[\mathrm{MSO}_{3}^{-}]}$ (S2)

At the same time, there are mass and charge balance of PILs in solution:

$[[\mathrm{RH}^{+}$][$\mathrm{MSO}_{3}^{-}$]]_total_ = [$\mathrm{MSO}_{3}^{-}$] = $[\mathrm{RH}^{+}$] + $[\left[ R^{-} \right]^{+}]$ (S3)

$\left[ Na^{+} \right]$ + $[\mathrm{RH}^{+}$] + $\left[ SH_{2}^{+} \right]$ = [$S^{-}$] + $\left[ \mathrm{MS}O_{3}^{-} \right]$ (S4)

In these equations, [X] stands for the concentration of each ion at any moment of titration. From equations S2, S3, and S4, we have the equation of *K*a:

$K_{a}=\frac{\left[ Na^{+} \right]+\left[ SH_{2}^{+} \right]-[S^{-}]}{\left[ S^{-} \right]+[\mathrm{MSO}_{3}^{-}]-\left[ Na^{+} \right]-\left[ SH_{2}^{+} \right]}*\left[ \mathrm{SH}_{2}^{+} \right]$ (S5)

When the titration was carried out at the pH value below 7 against a solution of 0.01 mol/L of PILs, there is an assumption that the value of [S^-^] could be neglected and the equation S5 becomes:

$K_{a}=\frac{\left[ Na^{+} \right]+\left[ SH_{2}^{+} \right]}{[\mathrm{MSO}_{3}^{-}]-\left[ Na^{+} \right]-\left[ SH_{2}^{+} \right]}*\left[ \mathrm{SH}_{2}^{+} \right]$ (S6)

In the equation S6, the only unknown value is [SH_2_^+^]. In order to get the value, the activity coefficient $f\left[ \mathrm{SH}_{2}^{+} \right]$ is introduced to calculation processes because it is relevant to [SH_2_^+^] through the following definition:

[SH_2_^+^] = $\frac{a\left[ SH_{2}^{+} \right]}{f\left[ \mathrm{SH}_{2}^{+} \right]}$ (S7)

In equation S7, $f\left[ \mathrm{SH}_{2}^{+} \right]$ could be calculated by activity coefficient formula:

-log$f\left[ \mathrm{SH}_{2}^{+} \right]$ = $\frac{\frac{1}{2}Z^{2}I^{\frac{1}{2}}}{1+I^{\frac{1}{2}}}$ (S8)

I = $\frac{1}{2}\Sigma Z^{2}\left[ X \right]$ (S9)

In the formula S8 and S9, Z represents the charge number of ions and [X] represents the concentration of ions. But what deserve our attention is that the ionic strength of solution is almost no difference in the course of titration because high-concentration KCl as solution. Therefore, the change of ionic strength of solution in the process of titration could be ignored.

**Figure S33 |** The titration curves of synthesized PILs (black dot line) and their first-order derivative (red dot line) at 25 ± 0.1 ℃.

1. The GC chromatogram of catalytic result for the conversion of cyclohexanol in the catalyst of PILs.

**Figure S34** **|** The GC chromatogram of catalytic result for the conversion of cyclohexanol in the catalyst of [MDEA][mesy]

n-dodecane

cyclohexanol

[cyclohexane](javascript:;)

n-dodecane

cyclohexanol

cyclohexane

**Figure S35** **|** The GC chromatogram of catalytic result for the conversion of cyclohexanol in the catalyst of [DEA][mesy]

n-dodecane

cyclohexane

n-dodecane

cyclohexane

**Figure S36** **|** The GC chromatogram of catalytic result for the conversion of cyclohexanol in the catalyst of [MDEA][OTf]

n-dodecane

cyclohexane

n-dodecane

cyclohexanol

cyclohexane

**Figure S37** **|** The GC chromatogram of catalytic result for the conversion of cyclohexanol in the catalyst of [DMEA][OTf]

n-dodecane

cyclohexanol

cyclohexane

**Figure S38** **|** The GC chromatogram of catalytic result for the conversion of cyclohexanol in the catalyst of [TEA][OTf]

n-dodecane

cyclohexene

**Figure S39** **|** The GC chromatogram of catalytic result for the conversion of cyclohexanol in the catalyst of [DEA][OTf]

n-dodecane

cyclohexanol

cyclohexene

cyclohexane

**Figure S40** **|** The GC chromatogram of catalytic result for the conversion of cyclohexanol in the catalyst of [MDEA][Bsa]

n-dodecane

cyclohexanol

cyclohexene

cyclohexane

**Figure S41** **|** The GC chromatogram of catalytic result for the conversion of cyclohexanol in the catalyst of [DMEA][Bsa]

n-dodecane

cyclohexanol

cyclohexene

cyclohexane

**Figure S42** **|** The GC chromatogram of catalytic result for the conversion of cyclohexanol in the catalyst of [TEA][Bsa]

n-dodecane

cyclohexanol

cyclohexene

cyclohexane

**Figure S43** **|** The GC chromatogram of catalytic result for the conversion of cyclohexanol in the catalyst of [DEA][Bsa]
